# Complete Sequence of pEC012, a Multidrug-Resistant IncI1 ST71 Plasmid Carrying *bla*_CTX-M-65_, *rmtB*, *fosA3*, *floR*, and *oqxAB* in an Avian *Escherichia coli* ST117 Strain

**DOI:** 10.3389/fmicb.2016.01117

**Published:** 2016-07-18

**Authors:** Yu-Shan Pan, Zhi-Yong Zong, Li Yuan, Xiang-Dang Du, Hui Huang, Xing-Hao Zhong, Gong-Zheng Hu

**Affiliations:** ^1^College of Animal Science and Veterinary Medicine, Henan Agricultural UniversityZhengzhou, China; ^2^West China Hospital, Sichuan UniversityChengdu, China

**Keywords:** extended-spectrum β-lactamases, 16S rRNA methylase, fosfomycin, Tn*2*, IS*26*-composite transposon

## Abstract

A 139,622-bp IncI1 ST71 conjugative plasmid pEC012 from an avian *Escherichia coli* D-ST117 strain was sequenced, which carried five IS*26*-bracketed resistance modules: IS*26*-*fosA3*-*orf1-orf2-Δorf3*-IS*26*, IS*26-fip*-ΔIS*Ecp1*-*bla*_CTX-M-65_-IS*903D*-*iroN*-IS*26*, IS*26-ΔtnpR*-*bla*_TEM-1_-*rmtB*-IS*26*, IS*26*-*oqxAB*-IS*26*, and IS*26*-*floR*-*aac(3)-IV*-IS*26*. The backbone of pEC012 was similar to that of several other IncI1 ST71 plasmids: pV408, pM105, and pC271, but these plasmids had different arrangements of multidrug resistance region. In addition, the novel IS*Ec57* element was identified, which is in the IS*21* family. The stepwise emergence of multi-resistance regions demonstrated the accumulation of different resistance determinants through homologous recombination. To the best of our knowledge, this is the first study to identify a multidrug-resistant IncI1 ST71 plasmid carrying *bla*_CTX-M-65_, *rmtB*, *fosA3*, *floR*, and *oqxAB* in an avian *E. coli* ST117 strain.

## Introduction

The emergence and dissemination of antimicrobial resistance have become a major global public health concern. It is associated with mobile genetic elements such as plasmids, transposons, and integrons. Homologous recombination is important in the movement of resistance genes and the creation and evolution of multiresistance region (MRR; Partridge, 2011; van Hoek et al., 2011). In the recent years, *bla*_CTX-Ms_ has become the most common genes encoding extended-spectrum β-lactamases in multidrug-resistant *Enterobacteriaceae* worldwide. The *bla*_CTX-Ms_ genes often coexist with other genes such as *armA* or *rmtB* encoding 16S rRNA methylases, or with *fosA3*, which confers resistance to fosfomycin (Deng et al., 2011; Hou et al., 2012). Recently, multiple resistance genes *bla*_TEM-1_, *bla*_CTX-M-65_, *fosA3*, and *rmtB* were found to coexist on the same IncFII plasmid such as F33:A-:B- plasmid pHN7A8, F33:A-:B- plasmid pEC011, and F2:A-:B- plasmid pXZ, which were obtained from *Escherichia coli* isolates originating from dogs, chickens and ducks in China, respectively (Sun et al., 2012; He et al., 2013; Pan et al., 2014). Of note, the similar IncFII plasmids carrying these genes were also identified in *E. coli* and *Klebsiella pneumoniae* from humans (Xiang et al., 2015; Zhao et al., 2015; Sennati et al., 2016). Moreover, plasmids belonging to IncI1 carrying extended-spectrum and AmpC β-lactamase genes are widespread in *Enterobacteriaceae* (Garcia-Fernandez et al., 2008). Recently, IncI1 ST71 epidemic plasmid lineage carrying *bla*_TEM-1_, *bla*_CTX-M-65_, *fosA3* was identified in *E. coli* from humans and animals (Yang et al., 2014; Riccobono et al., 2015). Here, we report the complete sequence of pEC012, a multidrug-resistant IncI1 ST71 plasmid carrying *bla*_TEM-1_, *bla*_CTX-M-65_, *rmtB*, *fosA3*, *floR*, and *oqxAB* in an avian *E. coli* ST117 isolate.

## Materials and Methods

### Bacterial Strains

In a survey on antimicrobial resistant bacterial strains in China in 2009, one *E. coli* strain, EC012, was isolated from one chicken on a farm in Changchun Province, Northeast China. The species identification was performed using the VITEK 32 automated identification system (bioMérieux, Marcy l’Etoile, France). *E. coli* ATCC 25922 was used as the control strain for the antimicrobial susceptibility testing.

### Antimicrobial Susceptibility Testing and Antimicrobial Resistance Gene Detection

Antimicrobial susceptibility testing was performed on EC012 using the broth microdilution method, and the minimal inhibitory concentration of fosfomycin was determined using the agar dilution method on Mueller–Hinton agar containing 25 μg/mL glucose-6-phosphate according to the guidelines of the Clinical and Laboratory Standards Institute (Clinical and Laboratory Standards Institute [CLSI], 2013). The presence of antimicrobial resistance genes encoding extended-spectrum β-lactamases, 16S rRNA methylases and the plasmid-encoded fosfomycin-resistance determinants were screened using PCR as described previously (Hou et al., 2012; Pan et al., 2013). The *floR* gene was amplified by primers (floR-F: 5′-GTATGGGCACCTTCTTCGTCT-3′ and floR-R: 5′- CAGCCCCAACGAAACCAGT-3′) in this study.

### Phylogenetic Group and Multilocus Sequence Typing (MLST)

Phylogenetic typing was done by means of a multiplex PCR-based method with the *chuA*, *yjaA* genes and the DNA fragment TSPE4.C2, as described previously (Clermont et al., 2000). Seven housekeeping genes (*adk*, *fumC*, *gyrB*, *icd*, *mdh*, *purA*, and *recA*) were amplified and sequenced as described previously (Wirth et al., 2006). The sequences were analyzed further by MLST according to the protocols recommended at http://mlst.warwick.ac.uk/mlst/dbs/Ecoli.

### Conjugation Experiment and Plasmid Analysis

Conjugation experiment was carried out using *E. coli* EC012 as the donor and *E. coli* C600 (resistant to rifampicin) as the recipient, as described previously (Pan et al., 2013). The transconjugants were selected on MacConkey agar supplemented with cefotaxime (4 μg/mL) and rifampicin (450 μg/mL). Transfer frequency was calculated as the number of transconjugants per recipient. Antimicrobial susceptibility testing of the transconjugants was conducted, and the presence of *bla*_CTX-M-65_, *rmtB*, *fosA3*, and *floR* was confirmed by PCR as described above. Plasmid DNA was extracted from the transconjugants using the Plasmid Midi Kit (Qiagen, Hilden, Germany), designated as pEC012. The plasmid incompatibility groups of pEC012 were typed by a PCR-based method, as described previously (Carattoli et al., 2005). Plasmid MLST was carried out using published primers with alleles (Garcia-Fernandez et al., 2008) and sequence types assigned according to http://pubmlst.org/plasmid.

### Plasmid Sequencing and Annotation

Plasmid pEC012 from the transconjugant TEC012 was fully sequenced by an Illumina Miseq platform. Pair-end index libraries were constructed using NEBNext Ultra DNA Library Prep Kit (Illumina, San Diego, CA, USA). Libraries with different indexes were mixed and loaded on an Illumina MiSeq. Sequencing was carried out using a 2 × 250 paired-end configuration on the Miseq instrument. Sequence data were assembled into eight contigs using Velvet 1.1.06 and CAP3 software (Huang and Madan, 1999; Zerbino and Birney, 2008). Gaps were closed by a PCR-based strategy. The plasmid sequence was initially annotated with the Rapid Annotation using Subsystem Technology (RAST version 2.0) server^1^ and then curated manually using the BLASTn and BLASTp algorithms^2^. The comparative analysis of complete nucleotide sequences was performed using the referenced plasmids IncI1 R64 (GenBank accession no. AP005147), IncI1 ST71 plamsid pC193, pM105, pV408, and pC271 (GenBank accession no. LN735558, LN735559, LN735560, LN735561, respectively; Sampei et al., 2010; Riccobono et al., 2015). Physical maps were generated using EasyFig software and DNAPlotter (Carver et al., 2009; Sullivan et al., 2011).

### Nucleotide Sequence Accession Number

The complete sequence of pEC012 has been deposited in the GenBank database under accession no. KT282968.

## Results and Discussion

### Strain and Plasmid Characteristics

The *E. coli* strain was shown to be resistant to ampicillin, cefotaxime, amikacin, gentamycin, fosfomycin, doxycycline, florfenicol, and ciprofloxacin (Supplementary Table S1), and carried *bla*_TEM-1_, *bla*_CTX-M-65_, *rmtB*, *fosA3*, and *floR* genes, belonging to D-ST117. *E. coli* EC012 strain was isolated from the same farm as *E. coli* D-ST117 EC011, which exhibit the same resistant phenotype (Pan et al., 2014).

The transconjugants were obtained successfully, designated as TEC012, exhibiting resistance to cefotaxime, amikacin, fosfomycin and florfenicol, and low-level resistance to ciprofloxacin (Supplementary Table S1). The conjugation frequency of pEC012 was 5 × 10^-3^. pEC012 belonged to the incompatibility group IncI1, which was further assigned to ST71, and carried *bla*_TEM-1_, *bla*_CTX-M-65_, *rmtB*, *fosA3*, and *floR* genes. However, the pEC011 from the strain EC011belonged to IncFII, and carried *bla*_TEM-1_, *bla*_CTX-M-65_, *rmtB*, *fosA3* genes, not harbored the *floR* gene (Pan et al., 2014).

### Overall Structure of pEC012

**FIGURE 1:**
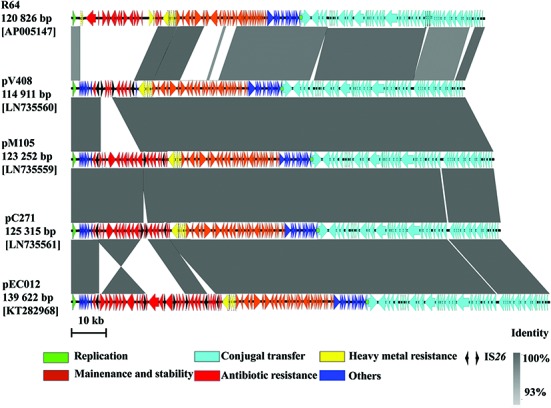
**Comparative analysis of pEC012 with other IncI1 plasmids.** Homologous segments generated by a BLASTn comparison (≥93% identity) are shown as gray boxes. Genes are represented by thick arrows. The location of oriT is indicated by a green dot. The color code equates to that described in this figure legend.

pEC012 is 139,622-bp in length with a GC content of 50.8%. The plasmid was composed of a 102,866-bp IncI1 typical backbone fragment encoding genes responsible for plasmid replication, transfer, maintenance, and stability functions, and a 36,756-bp MRR (**Supplementary Figure S1**). The backbone of pEC012 was similar to other IncI1 ST71 plasmids such as pV408, pM105, and pC271 (>99% at nucleotide level; **Figure 1**). At least 156 complete open reading frames were predicted within the plasmid.

### pEC012 MRR

**FIGURE 2:**
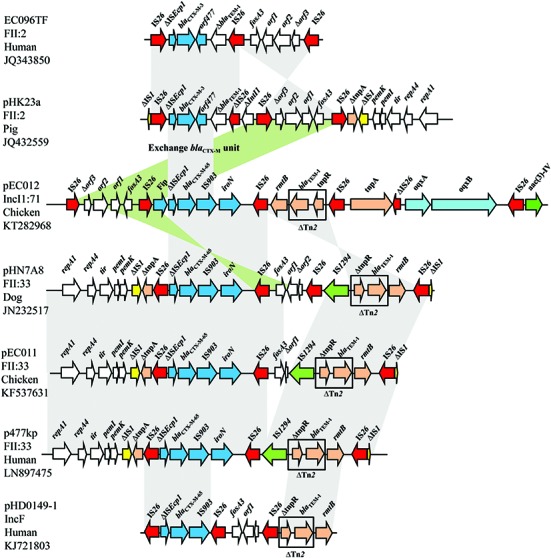
**Structural features surrounding the *bla*_CTX-M-65_ gene in pEC012 compared with other *bla*_CTX-M_ of IncFII-type plasmids EC096TF (GenBank accession number JQ343850), pHK23a (GenBank accession number JQ432559), pHN7A8 (GenBank accession number JN232517), pEC011 (GenBank accession number KF537631), p477kp (GenBank accession number LN897475), pHD0149-1 (GenBank accession number KJ721803).** Similar regions are indicated by gray shading.

The MRR of pEC012 had complicated structure containing several transposable units with a new arrangement. It consisted of five different segments containing resistance genes. The first segment corresponded to the IS*26*-formed composite transposon carrying the *fosA3* gene, conferring resistance to fosfomycin. The segment IS*Ecp1*-*bla*_CTX-M-65_-IS*903*-*iroN* was the second transposable unit, in which IS*Ecp1* was truncated by *fip* gene. The third segment, including *rmtB* adjacent to a fragment of Tn*2* carrying *bla*_TEM-1_, was linked with the second segment by IS*26* (**Figure 2**).

Recently, the IncI1 ST71 epidemic plasmid carrying IS*26*-*fip*-ΔIS*Ecp1*-*bla*_CTX-M-65_-IS*903D*-*iroN*-IS*26* and IS*26*-*fosA3*-*Δorf1*-IS*26* modules was reported in the Chaco region of Bolivia, which lacked the *tnpR*-*bla*_TEM-1_-*rmtB* module (Riccobono et al., 2015). In addition, the IncF plasmids carrying *bla*_TEM-1_, *bla*_CTX-M-65_, *fosA3*, and *rmtB* have been found in *E. coli* isolates from animals (Sun et al., 2012; He et al., 2013; Pan et al., 2014), and in *E. coli* and *K. pneumoniae* from humans (Xiang et al., 2015; Zhao et al., 2015; Sennati et al., 2016). Those plasmids belonging to IncFII possessed a similar MRR structure (**Figure 2**). In the present study, the IS*26*-*fosA3*-IS*26* module was located upstream of the IS*Ecp1*-*bla*_CTX-M-65_-IS*903*-*iroN* module, and Tn*2* carrying *rmtB* was downstream of the IS*Ecp1*-*bla*_CTX-M-65_-IS*903*-*iroN* module in the opposite orientation with other MRRs of IncF plasmids, such as pHN7A8, pEC011, p477kp, and pHD0149-1 (GenBank accession no. JN232517, KF537631, LN897475, KJ721803, respectively; **Figure 2**). Seven copies of IS*26* were dispersed in the MRR of pEC012, however, target duplication repeats flanking IS*26* were not observed. The lack of direct repeats flanking IS*26* in the pEC012 MRR suggests that this may have occurred by homologous recombination rather than insertion (Partridge, 2011).

Moreover, pEC012 belonged to IncI1, whereas pEC011 belonged to IncFII, suggesting that the complex MRR was mobilized into different plasmids. However, it was difficult to discriminate if the MRR carrying *bla*_TEM-1_, *bla*_CTX-M-65_, *fosA3*, and *rmtB* was transferred from IncFII to IncI1, or vice versa.

**FIGURE 3:**
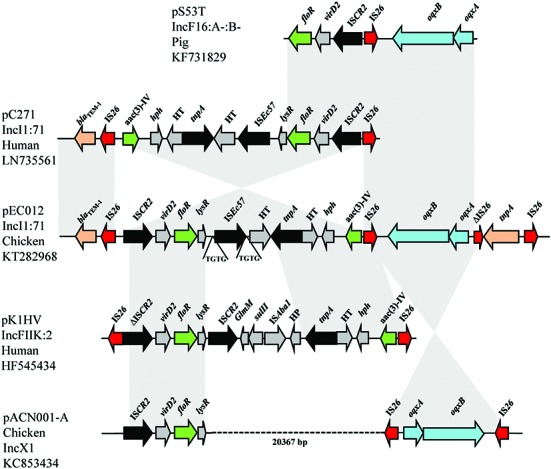
**Structural features of IS*26* composite transposon carrying *floR*, *aac(3)-IV*, and *oqxAB* in pEC012 compared with those of IncFII plasmid pS53T (GenBank accession number KF731829), IncI1 pC271 (GenBank accession number LN735561), IncFIIK pK1HV (GenBank accession number HF545434), and the IncX1 pACN001-A (GenBank accession number KC853434).** Similar regions are indicated by gray shading.

Two IS*26* composite transposons located downstream of Tn*2* transposon carrying *rmtB* were also observed. One IS*26* composite transposon harbored *oqxAB*, and another contained *floR* and *aac(3)-IV* genes (**Figure 3**). The typical context of *floR* is IS*CR2*-*virD2*-*floR*-*lysR* (Liu et al., 2014; Wang et al., 2014), which could coexists with IS*26*-*oqxAB*-IS*26*, or *aac(3)-IV* in the other plasmids, such as pS53T, pC271, pK1HV, and pACN001-A (GenBank accession no. KF731829, LN735561, HF545434, KC853434, respectively). In the present study, the IS*26*-formed composite transposon harboring *floR* and *aac(3)-IV* was identical to that on pC271 and pM105 plasmids except for the direction (**Figure 1**). Nevertheless, no direct repeats flanking IS26 were found. This suggests that two copies of IS*26* could form a composite transposon and generate recombination. The IS*26*-formed composite transposon may have played an important role in generation of this complicated MRR by homologous recombination, and demonstrated the accumulation of different resistant determinants justly.

### Identification of IS*Ec57*, a Novel IS

IS*Ec57* is 1960-bp long and belongs to the IS*21* family^3^. It possesses 23/27-bp inverted repeats, and creates 4-bp directly repeated sequences of the target site (TGTG in the structure identified; **Figure 3**). IS*Ec57* contains two open reading frames (Orf1 and Orf2) encoding proteins of 341 and 257 amino acids, respectively. The deduced amino acid sequences of the Orf1 and Orf2 proteins had 87 and 94% amino acid identity with the transposase subunits of IS*Aba8*, respectively.

## Conclusion

We characterized the complete sequence of pEC012, a multidrug-resistant IncI1 ST71 plasmid carrying several resistance determinants, *bla*_TEM_, *bla*_CTX-M-65_, *rmtB*, *fosA3*, *oqxAB*, *floR*, and *aac(3)-IV* in an avian *E. coli* D-ST117 strain. The stepwise emergence of the MRR demonstrated the accumulation of different resistant determinants. IS*26* may play a pivotal role in generation of the complex genetics of resistance genes by homologous recombination. The detection of several resistance determinants on a conjugative plasmid among *E. coli* of food-producing animal origin may represent an emerging threat to animal and public health. There is an urgent need to monitor the dissemination of this multidrug-resistance plasmid among the *Enterobacteriaceae*.

## Author Contributions

Y-SP is in charge of design of study, acquisition and analysis of data, drafting of article; Z-YZ is in charge of analysis of data and critical revision; LY is in charge of analysis of data and drafting of article; X-DD is in charge of drafting of article; HH is in charge of acquisition of data; X-HZ is in charge of acquisition of data; G-ZH is in charge of conception and design of study, and analysis of data.

## Conflict of Interest Statement

The authors declare that the research was conducted in the absence of any commercial or financial relationships that could be construed as a potential conflict of interest.

## Footnotes

1 http://rast.nmpdr.org

2 http://blast.ncbi.nlm.nih.gov/blast

3 http://www-is.biotoul.fr/

## Supplementary material

FIGURE S1 **Genetic map of multi-resistance plasmid pEC012.** Coding regions are shown indicating the direction of transcription. The different plasmid modules are indicated on the inner circle of the map. The backbone of the plasmid consists of a replication, maintenance and stability (green), conjugal transfer module (gray). The MRR of the plasmid is composed of a 36.7-kb region (red).
