# Supplementary material for: Complete Sequence of pEC012, a Multidrug-Resistant IncI1 ST71 Plasmid Carrying blaCTX-M-65, rmtB, fosA3, floR, and oqxAB in an Avian Escherichia coli ST117 Strain
Source: Front Microbiol. 2016 Jul 18;7:1117. doi: 10.3389/fmicb.2016.01117 (PMC4947595; doi:10.3389/fmicb.2016.01117)
Supplement: Supplementary file 2 [file Table_1.DOC]

**Table S1.** Properties of the *E. coli* EC012, transconjugant TEC012, recipients *E. coli* C600, and quality control strain *E. coli* ATCC 25922 in this study

| Isolate | Phylogroup | MLST | MIC (μg/mL) | | | | | | | | | |
| --- | --- | --- | --- | --- | --- | --- | --- | --- | --- | --- | --- | --- |
|  |  |  | AM | CTX | GEN | KAN | AMK | ENR | CIP | DOX | FFC | FOS |
| EC012 | D | 117 | >256 | 128 | >256 | >256 | >256 | 64 | 32 | 64 | 256 | >256 |
| TEC012 |  |  | >256 | 64 | >256 | >256 | >256 | 0.5 | 0.5 | 1 | 128 | >256 |
| *E. coli* 600 |  |  | 4 | <0.125 | <0.125 | <0.125 | <0.125 | <0.125 | <0.125 | 0.25 | 2 | 1 |
| *E. coli* ATCC 25922 |  |  | 4 | <0.125 | <0.125 | <0.125 | <0.125 | <0.125 | <0.125 | 1 | 1 | 1 |

Note: AM, ampicillin; AMK, amikacin; CIP, ciprofloxacin; CTX, cefotaxime; DOX, doxycycline; ENR, enrofloxacin; FFC, florfenicol; FOS, fosfomycin; GEN, gentamicin; KAN, kanamycin. The MIC of antimicrobial drugs was determined by the broth microdilution method, except for fosfomycin, which was determined by the agar dilution method according to CLSI guidelines.
